# Functional multi-band THz meta-foils

**DOI:** 10.1038/srep03531

**Published:** 2013-12-18

**Authors:** Jianfeng Wu, Herbert O. Moser, Su Xu, Linke Jian, Agnieszka Banas, Krzysztof Banas, Hongsheng Chen, Andrew A. Bettiol, Mark B. H. Breese

**Affiliations:** 1 Center for Ion Beam Applications (CIBA), Department of Physics, National University of Singapore, 2 Science Drive 3, 117542, Singapore; 2 Karlsruhe Institute of Technology (KIT), Network of Excellent Retired Scientists (NES) and Institute of Microstructure Technology (IMT), Postfach 3640, 76021 Karlsruhe, Germany; 3 The Electromagnetics Academy at Zhejiang University, Department of Information Science & Electronics Engineering, Zhejiang University, Hangzhou 310027, China; 4 Singapore Synchrotron Light Source (SSLS), National University of Singapore, 5 Research Link, 117603, Singapore

## Abstract

In this paper, we present the first experimental demonstration of double- and triple-band negative refraction index meta-foils in the terahertz (THz) region. Multi-band meta-foils constructed by multi-cell S-string resonators in a single structure exhibit simultaneously negative permittivity and negative permeability responses at multiple frequencies. The phenomena are confirmed by numerical simulations and Fourier transform infrared spectroscopy measurements. The flexible, freestanding multi-band meta-foils provide a promising candidate for the development of multi-frequency THz materials and devices.

Electromagnetic metamaterials as theoretically introduced by Veselago^1^ and experimentally demonstrated by Pendry, Smith, and others^2^,^3^ are artificially structured media which can interact with and control electromagnetic waves. They possess novel electromagnetic properties, such as simultaneously negative permittivity and permeability^1^,^2^,^3^. These enable many functional applications including negative refraction^3^,^4^,^5^, superlensing^6^,^7^, and invisibility cloaking^8^,^9^,^10^,^11^. Natural materials only exhibit a weak magnetic response at terahertz frequencies in the range of 0.1 to 10 THz, which covers the so-called “THz gap”^12^. This remains a little developed portion of the electromagnetic spectrum due to the lack of efficient sources, detectors and functional devices. Metamaterials, unlike natural materials, can be artificially tailored to exhibit strong resonant behavior at terahertz frequencies^13^,^14^,^15^,^16^,^17^,^18^,^19^,^20^,^21^. As a result, functional THz metamaterial devices, such as switches^17^,^18^, filters^19^, modulators^20^, and perfect absorbers^21^, have been investigated.

THz meta-foils, proposed by Moser *et al.*, are a new three-dimensional left-handed metamaterial, which are based on the interconnected ‘S-string’ architecture^22^,^23^,^24^,^25^,^26^. A self-supported, locally stiff, globally flexible space-grid is formed by the upright S-string architecture with the distinctive feature of metallic transverse interconnects. Meta-foils are all-metal, self-supported, free-standing electromagnetic metamaterials. Their properties are solely determined by the geometric structure and the metal properties due to no dielectrics such as embedding matrices or supporting substrates. Seemingly similar structures were presented by the name of ‘meander’^27^, however, those meander structures are fundamentally different from meta-foils as they lack the spatial phase shift by half S length between adjacent S-strings introduced by Chen *et al*^22^*.*, who did not consider meander structures for their obvious lack of strong resonators. This structural difference was discussed by Moser *et al*^25^*.* to entail a 3000fold higher resonance peak in the shifted strings. Meta-foils can be considered as being composed of identical inductance-capacitance unit cells which are galvanically coupled to each other in both directions of a plane array. Meta-foils exhibit a magnetic resonance peak in transmission which supports electromagnetic waves propagating through them. Previous meta-foils were based on single cells featuring one magnetic resonance peak observed in transmission at frequencies of typically 3–4 THz. Since the magnetic resonance is fixed by the geometrical parameters of resonator unit cell, meta-foils with multiple cells and resonances become possible, much in analogy to the work done in the GHz range by Chen *et al*^28^.

In this paper, we extend our research from conventional single-band meta-foils to multi-band meta-foils in the THz region. Multi-band meta-foils constructed by multi-cell S-string resonators in a single structure exhibit simultaneously negative permittivity and negative permeability responses at multiple frequencies. We demonstrate the fabrication of multi-band meta-foils, study their resonance properties and outline potential applications. The simple pattern and the flexible multi-band exhibited indicate the superiority of the multi-cell meta-foils in the future development of multi-frequency THz materials and devices.

## Results

In this work, the simulations of meta-foils' properties were carried out by using the frequency domain solver of the CST Microwave Studio, which implemented a finite element method to determine reflection and transmission properties. In the simulations, the unit cell boundary condition was applied, and the gold was modeled as a lossy metal^29^ with conductivity σ = 4.09 × 10^7^ Sm^−1^.

**Figure 1:**
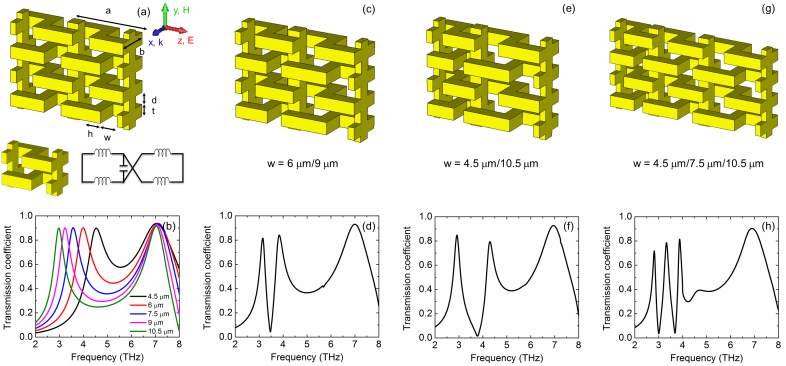
Functional multi-band THz meta-foils designs and simulated results. (a) and (b) 3D schematic and simulated transmission spectra of individual single-cell meta-foils with different S-string length at normal incidence. The resonant unit cell and its equivalent circuit diagram are also depicted. All geometric parameters are given by a = 2w + 3h, b = 15 μm, h = t = d = 5 μm, w is varied from 4.5 to 10.5 μm. The electric field vector E points in z-direction, i.e., along the S-strings, and the magnetic field vector H points in y-direction, i.e., perpendicular to the resonance loops. The magnetic resonance frequency changes from 4.53 over 3.98, 3.57, 3.23 to 2.97 THz as the open width w changes from 4.5 μm to 10.5 μm. (c)–(f) 3D schematics and simulated transmission spectra of bi-cell meta-foils. The open width w is alternated between 6 μm and 9 μm for (c), and 4.5 μm and 10.5 μm for (e). Two magnetic resonance peaks are at 3.15 THz and 3.83 THz for (d), and at 2.92 THz and 4.29 THz for (f). (g) and (h) 3D schematic and simulated transmission spectrum of tri-cell meta-foils (w = 4.5 μm/7.5 μm/10.5 μm). Three magnetic resonances are at 2.84 THz, 3.36 THz, and 3.89 THz.

As shown in Fig. 1, all individual S-strings are connected by transverse rods creating a space-grid to be a meta-foil which is self-supported, locally stiff, and globally flexible. These connections are made between the oscillation nodes of current in the strings to minimize any influence on resonances. Single-cell meta-foils that feature the same unit cell across the whole meta-foil area are shown in Fig. 1a. The unit cell includes two inductance loops that are connected to the same capacitor, which is picturesquely described by the equivalent circuit. An inductance loop is formed by two bars, one lying in the upstream plane and the other one in the downstream plane, that are connected by the interconnecting line. The capacitor is formed by the two b-legs belonging to adjacent S-strings that are connected to the two bars. Figure 1b shows the transmission spectra of single-cell meta-foils. Two dominant resonance peaks appear in each transmission spectrum. The peak at lower frequency is a left-handed magnetic resonance, and the other one at higher frequency is a right-handed electrical resonance, which is confirmed by the parameter retrieval calculations and electric field distribution below. Here, the S-string length is defined by a = 3h + 2w where h is the width of conducting bars and w is the open width of each U portion (there are two ‘U’ motifs in each S). When the S-string length increases, the magnetic peak is red-shifted. The magnetic resonance exhibits a red shift from 4.53 THz to 2.97 THz when the resonator length is increased from 24 μm to 36 μm, which can be explained by the simplified LC oscillator model. Actually meta-foils can be described by a network of coupled LC oscillators. The magnetic resonance frequency is proportional to 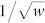 because the inductance L scales linearly with the open width w whereas the capacitance formed by the two opposite b-legs of two adjacent S-strings remains unchanged. In comparison, the electrical peak remains the same because a variation of w increases/decreases the respective capacitance and inductance in opposite directions, resulting in no net change. Note that the capacitance acting in case of the electrical resonance is different from the above as it is formed by the two subsequent b-legs in the same S-string. So, if the length of the S-structure is increased, the inductance raises, but the capacitance decreases resulting in little change of the resonance frequency. In summary, the magnetic resonance properties of meta-foils can be tuned by changing the length of the resonator cell.

Starting from these results of single-cell meta-foils, we propose meta-foils featuring two or three single cells with different dimensions in the same structure to realize a bi-cell or tri-cell meta-foils with two or three distinct magnetic resonances. Bi-cell and tri-cell meta-foils in which the open width w alternates between two or three different values are shown in Figs. 1c to h. Two bi-cell meta-foils (w = 6 μm/9 μm and w = 4.5 μm/10.5 μm) are displayed in Figs. 1c and e. In the simulated transmission spectra, two separate magnetic resonances are excited at 3.15 THz and 3.83 THz for bi-cell meta-foils with w = 6 μm/9 μm, and at 2.92 THz and 4.29 THz where w = 4.5 μm/10.5 μm. These two distinct left-handed magnetic resonances appear due to the superposition of the different resonances formed by each individual cell size of corresponding single-cell meta-foils as well as the mutual coupling between different cells in the bi-cell meta-foils. Their individual peak positions in Figs. 1d and f are close to those of the corresponding single-cell meta-foils in Fig. 1b, except for a slight red shift (stronger for the higher frequency peak). Furthermore, we note that the resonance peaks do not simply superpose, in contrast, a deep dip develops almost down to zero between them. This is due to the opposite phase of two oscillators in between resonance peaks. In the same manner, Figures 1g and h display 3D schematic and corresponding simulated spectra for tri-cell meta-foils (w = 4.5 μm/7.5 μm/10.5 μm). In the tri-cell meta-foils, three magnetic resonances at 2.84 THz, 3.36 THz and 3.89 THz are strongly excited.

**Figure 2:**
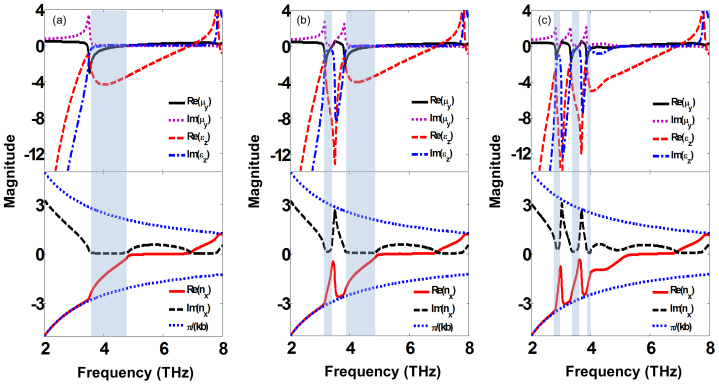
Retrieved parameters of multi-band THz meta-foils. Three different types of meta-foils, single-cell meta-foils (w = 7.5 μm) for (a), bi-cell meta-foils (w = 6 μm/9 μm) for (b), and tri-cell meta-foils (w = 4.5 μm/7.5 μm/10.5 μm) for (c), were calculated from the simulated transmission at normal incidence. In the shaded frequency ranges, both the permittivity and permeability are negative.

As we did previously on 1SE single-cell meta-foils^23^,^25^, we run a standard parameter retrieval code^30^ on these spectral data with the sole purpose to check for a negative refractive index in the magnetic resonance peaks of the bi- and tri-cell meta-foils in comparison with the single-cell meta-foils. The retrieval results are shown in Fig. 2 where the frequency bands with negative refractive index are highlighted. It is clearly seen that for all double and triple resonances, the refractive index is negative. In particular, the tri-cell meta-foils exhibit three left-handed bands which have not been achieved in THz single structure before. The retrieval results in Fig. 2 show that, from single- over bi- to tri-cell meta-foils, the number of negative refractive index peaks corresponds to the number of different cells and they meet the respective frequency bands. Negative index peaks are located at the magnetic resonances. Overall, multiple-cell meta-foils are theoretically demonstrated to possess multiple magnetic resonances here. In some frequency ranges, Re(n) reaches the edge of the Brillouin zone which is defined as ±π/(kb). The wavelength inside of the medium becomes smaller than the thickness of the unit cell which renders the notion of refractive index meaningless. A thorough discussion of conditions necessary to extract meaningful effective parameter tensors from anisotropic thin slabs of metamaterials can be found in previous literatures^31^,^32^,^33^. Because of λ/b = 5 at 4 THz, b < λ/2 is fulfilled even at the highest frequency considered, where λ is the wavelength in free space which permeates the meta-foil. The limitations discussed by^31^,^32^,^33^ should not apply. The retrieval parameters provide a qualitatively explanation of the negative refractive index pass bands at the magnetic resonances.

**Figure 3:**
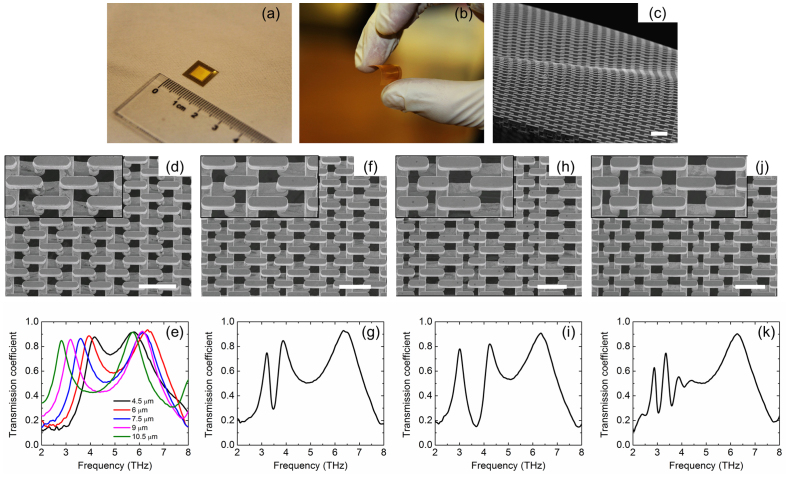
Experimental demonstration of multi-band THz meta-foils. (a) and (b) Photographs of the flat and bent meta-foils. The useful window is 6 mm × 6 mm × 0.015 mm (L × W × H). (c) SEM image of flexible meta-foils. (d) and (e) SEM image and measured transmission spectra of single-cell meta-foils. The measured magnetic resonance frequency changes from 4.18, 3.94, 3.60, 3.20 to 2.82 THz as the open width w changes from 4.5 μm to 10.5 μm. (f)–(i) SEM images and measured transmission spectra of bi-cell meta-foils. Two measured magnetic resonance peaks are at 3.11 THz and 3.77 THz for bi-cell meta-foils (w = 6 μm/9 μm), and at 2.90 THz and 4.13 THz for bi-cell meta-foils (w = 4.5 μm/10.5 μm). (j) and (k) SEM image and measured transmission spectra of tri-cell meta-foils (w = 4.5 μm/7.5 μm/10.5 μm). Three measured magnetic resonance peaks are at 2.87 THz, 3.35 THz and 3.87 THz. All scale bars in SEM images are 25 μm, and each inset shows a unit cell for each of the structures.

Figure 3 shows images and measured transmission spectra of single-cell, bi-cell and tri-cell meta-foils compared with Fig. 1. Figures 3a–c show the flat and deliberately bent meta-foils where the useful window is 6 mm × 6 mm × 0.015 mm (L × W × H). Single-cell meta-foils with different open width w from 4.5 μm to 10.5 μm are fabricated here, and a scanning electron microscopy (SEM) image of single-cell meta-foils (w = 7.5 μm) is selected to show in Fig. 3d. Figure 3e shows the measured transmission spectra of these different single-cell meta-foils at normal incidence. The measured magnetic resonance frequency changes from 4.18, 3.94, 3.60, 3.20 to 2.82 THz as the open width w changes from 4.5 μm to 10.5 μm. Compared with the simulated results in Figure 1b, the magnetic resonances are well reproduced over a substantial range of widths w, whereas the measured electric resonances are all at lower frequency than the simulated value and have a larger variation. Figures 3f to i depict SEM images of bi-cell meta-foils (w = 6 μm/9 μm and w = 4.5 μm/10.5 μm) along with their corresponding measured transmission spectra. For two different bi-cell meta-foils, two distinct magnetic resonances are obtained, at 3.11 and 3.77 THz where w = 6 μm/9 μm, and at 2.90 and 4.13 THz where w = 4.5 μm/10.5 μm. The positions of magnetic resonance peaks in the measured results are in good agreement with simulated results in Figs. 1d and f, demonstrating our ability to accurately fabricate bi-cell meta-foils. Remaining discrepancies between the experimental and simulated results in the positions, heights and widths of the resonance peaks are considered as being mainly due to small imperfections in both the fabrication process and characterization, which were ignored in simulations. Figures 3j and k show an SEM image of tri-cell meta-foil (w = 4.5 μm/7.5 μm/10.5 μm) along with its measured transmission spectrum. Three separate magnetic resonances are excited at 2.87 THz, 3.35 THz and 3.87 THz. The stronger reduction of the measured peak heights compared to the simulated ones as observed in single-cells and bi-cells is because the measured spectral resolution is no longer high enough, especially for the shaper resonance peak. The measured spectral resolution is 0.06 THz whereas the full width at half maximum (FWHM) of the high-frequency peak in the tri-cell is about 0.2 THz. So, only three measured points have to represent the sharp peak, thus touching at the limit set by Shannon's theorem.

**Figure 4:**
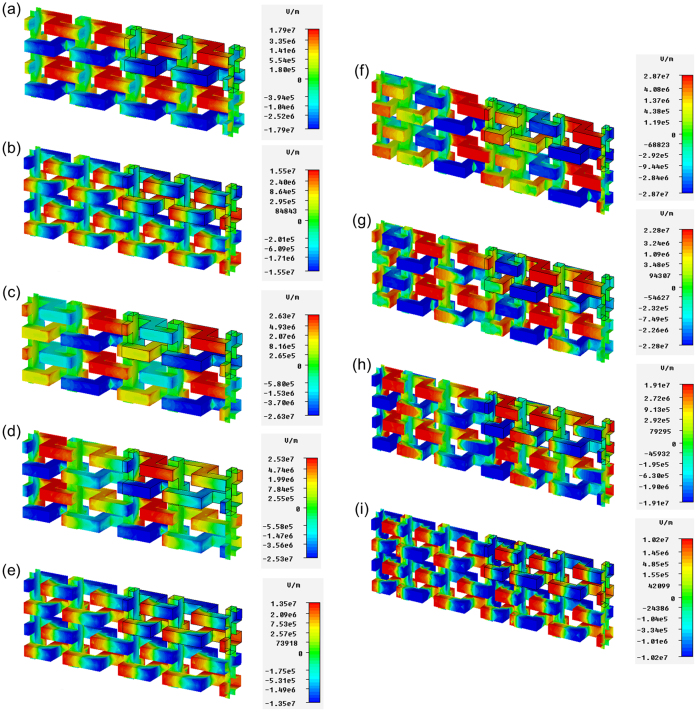
Electric field distributions of multi-band THz meta-foils at different resonances. (a) and (b) MWS simulation results of the electric field distribution on single-cell meta-foils (w = 7.5 μm) at 3.57 THz and 7.12 THz. (c)–(e) MWS simulation results of the electric field distribution on bi-cell meta-foils (w = 6 μm/9 μm) at 3.15 THz, 3.83 THz and 7.02 THz. (f)–(i) MWS simulation results of the electric field distribution on tri-cell meta-foils (w = 4.5 μm/7.5 μm/10.5 μm) at 2.84 THz, 3.36 THz, 3.89 THz and 7.05 THz.

In order to further understand the performance of single-, bi- and tri-cell meta-foils, we investigated the electric field distribution through numerical MWS simulation. Figures 4a and b show the electric field distribution in single-cell meta-foils (w = 7.5 μm) at magnetic and electrical resonances, respectively. It provides a more visual confirmation of the magnetic resonance at 3.57 THz and electrical resonance at 7.12 THz. At 3.57 THz, the adjacent S-strings are greatly excited with opposite field indicating strong charging of the capacitors formed by the b-legs of adjacent S-strings, and, thus, high amplitudes of oscillations in the LC loops creating the magnetic resonance. Note that in this magnetic resonance case the individual S-strings are at approximately the same potential level with adjacent strings at the opposite. In contrast, at 7.12 THz, each U portion in an S-string is electrically excited with some capacitive charging also between b-legs of the same S-string in addition to the potential differences between adjacent strings. Figures 4c and d show the electric field distribution in the bi-cell meta-foils (w = 6 μm/9 μm) at the magnetic resonances. Figure 4e displays the electric field distribution at the electrical resonance. At 3.15 THz and 3.83 THz, cells with w = 9 μm and w = 6 μm are strongly excited, respectively. At 7.02 THz, electric excitation similar to Fig. 4b is observed. Thus, in the bi-cell meta-foils the two magnetic resonances are selectively excited in either of the two single-cell sub-meta-foils. Figures 4f to i show the electric field distribution in the tri-cell meta-foils (w = 4.5 μm/7.5 μm/10.5 μm) at resonances. Similar to the bi-cell case, in tri-cell meta-foils three magnetic resonances are selectively excited in either of the three single-cell sub-meta-foils. If we compare these magnetic modes with those of other metamaterial structures, such as the split-ring resonator^3^, Ω-ring resonator^34^, subwavelength dielectric resonator^35^ and fishnet structures^36^,^37^,^38^, etc, we find they share similar magnetic mode in the frequency range of negative permeability. Although the shapes of these structures are quite different, the overall field distributions are all very similar. One can also see that the negative permeability vanishes when this magnetic mode disappears, e.g. 7.12 THz in the single-cell meta-foils, 7.02 THz in the bi-cell meta-foils and 7.05 THz in the tri-cell meta-foils. These results also imply that the proper magnetic modes in subwavelength scale are very important to achieve left-handed behavior.

## Discussion

The proposed multi-band meta-foils are different from previous work on THz dual-band electrical resonances in coupled split-ring resonators (SRRs)^39^,^40^,^41^,^42^,^43^,^44^,^45^,^46^,^47^,^48^ in a few crucial aspects. Multiple-cell meta-foils are characterized by galvanic coupling, magnetic excitation and transmission peaks at resonances whereas previous coupled SRR samples exhibit capacitive-inductive coupling, electric excitation and transmission dips at resonances, respectively. The continuous geometry of meta-foils results in stronger galvanic coupling compared to capacitive-inductive coupling in discrete SRR structures. In addition, no matter what multiple cells are designed into an actual meta-foil, the filling of the area is always complete in contrast to the <100% filling by split rings resonators. These fundamental differences make multiple-cell meta-foils a distinct class of metamaterials with unique characteristics.

Such multiple-cell meta-foils open up a wide range of new potential applications. In general, devices may exploit polychromatic properties or the coupling of spectro-geometric features. Among the former, multiple-passband filters, multi-band sensors, polychromators, and polychromatic beams may be envisaged, among the latter phase gratings and diffractive optical elements (DOEs). Applications would include spectral calibration and identification of molecules by multi-band filters, fast parallel sampling spectroscopy by polychromators, and metrology by phase gratings. Interestingly, multi-band meta-foils can also be mass manufactured cost-effectively to serve as optical elements by plastic moulding. To identify molecules, for instance, that exhibit absorption peaks in the THz range under discussion, they could be detected by means of a specific multi-band meta-foil that is designed to have transmission peaks at the spectral position of a selected molecule's absorption peaks. So, if broadband THz radiation is shone through a specific meta-foil having transmission resonances at 2.05, 2.9, 4.35 THz, the latter would act as a filter letting pass only the radiation corresponding to these resonances. If this filtered radiation would then pass through a sufficiently dense accumulation of the PETN molecule that features absorption peaks at these frequencies, it would be absorbed and the change in intensity could be measured by a simple broad-band detector. For TNT at 2.2, 3.8, 4.7 THz, it would work similarly. PETN (pentaerythritol tetranitrate) and TNT (2,4,6-trinitrotoluene) are well-known explosives having strong absorption bands at the above frequencies^49^, respectively. So, absence of the selected molecule or presence of other molecules would entail a higher detector response whereas the presence of the selected molecule would diminish detector response. The obvious potential application is in the detection of explosives in security. A broader discussion was given in^40^. Furthermore, interestingly, multi-band meta-foils can also be mass manufactured cost-effectively to serve as optical elements by plastic moulding^25^.

In conclusion, novel single-cell, bi-cell and tri-cell meta-foils have been manufactured and investigated. Results obtained from numerical simulation and spectroscopic characterization show that bi/tri-cell meta-foils feature two/three slightly red-shifted resonance peaks of the corresponding single-cell meta-foils which, however, are separated by a deep dip. The spectral location of each peak can be controlled by the geometric parameters, thus, the peaks are structurally tunable. In this way, multiple-cell meta-foils can be adapted to specified spectral signatures of target molecules. The concept might be extended to multi-cell meta-foils having four or more different cells with potential applications to novel multi-frequency devices, and, when grouping resonators of the same frequency, to fast polychromators or demultiplexers. In addition, multi-band meta-foils can be tailor-made to virtually any shape, they can be bent, and wrapped around objects to shield them from electromagnetic radiation, thus becoming true metamaterials on curved surfaces. Although implemented here at THz frequencies, multiple-cell meta-foils can also be extended to higher frequency infrared and optical frequencies.

## Methods

**Figure 5:**
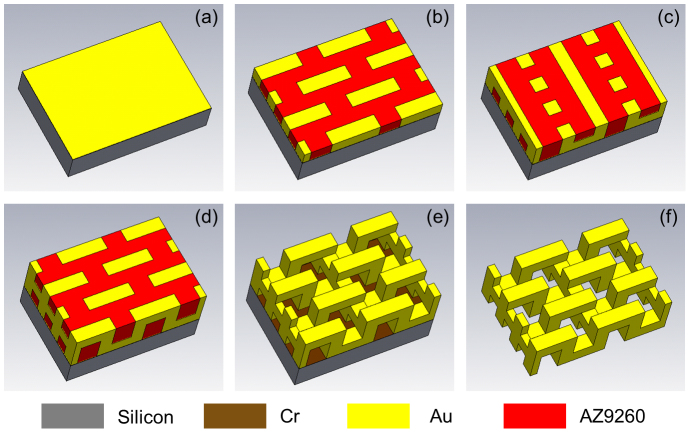
Flowchart of the fabrication of meta-foils. (a) Cr/Au (100 nm/50 nm) layers sputtered on silicon substrate. (b)–(d) Fabrication of three-layer structures by UV lithography and gold electroplating. (e) Removing AZ9260 photoresist and Au plating base step by step by acetone and gold etchant. (f) Releasing the whole structures from substrate by Cr etching.

Figure 5 depicts the fabrication process of meta-foils, using three levels of UV lithography with precise alignment and three repeated gold electroplating steps with accurate thickness control. Three optical masks were needed, which carried design patterns and alignment marks. For the initial UV photomask, a soda lime blank (Nanofilm, Wetlake Village, Califormia) with 100 nm thick chromium and 530 nm thick layer of AZ1518 photoresist was patterned by a direct-write laser system (Heidelberg Instruments uPG 101). A 500 μm thick silicon wafer was cleaned and covered with thin layers of Cr/Au (100 nm/50 nm) as an adhesion and plating base, respectively. A 5 μm thick AZ9260 resist was deposited by spin coating and then exposed by UV light in a Mask & Bond Aligner (Karl Suss, MA8/BA6). After resist development, the remaining resist mold was used for gold electroplating to build up a 5 μm thick gold layer. After gold electroplating, another thin layer of Au (50 nm) was sputtered on the sample as a new plating base for the fabrication of second layer structures. The same process sequence was repeated to obtain the second and third layers of the three-dimensional structure to give a total thickness of 15 μm. Precise alignment of photomasks with respect to the already processed structure on the substrate was critical during this process. Next, AZ9260 resist and Au plating base were removed step by step by acetone and gold etchant. Finally, the whole structure was released from substrate by Cr etching.

Bruker IFS 66 v/S FTIR was used to characterize the electromagnetic response of the meta-foils in the range of 2 to 8 THz. The radiation source was far infrared synchrotron radiation from the Infrared Spectro/Microscopy (ISMI) beamline at Singapore Synchrotron Light Source (SSLS)^50^. Transmission spectra were acquired by a PE/DLa TGS D201 detector, comparing signals measured with and without sample using a spectral resolution of 0.06 THz (2 cm^−1^). The sample chamber pressure was maintained at 2 mbar of dry nitrogen gas to avoid signal interferences from water vapor and other gases.

## Author Contributions

J.F.W. and L.K.J. designed the structures. J.F.W. performed the experiments. J.F.W. and H.O.M. wrote the manuscript. J.F.W., A.B. and K.B. performed FTIR measurements. S.X. and H.S.C. conducted the parameter retrieval calculations. M.B.H.B., H.O.M. and A.A.B. supervised the research. All the authors discussed the results and reviewed the manuscript.
